# A novel TLR7 agonist as adjuvant to stimulate high quality HBsAg-specific immune responses in an HBV mouse model

**DOI:** 10.1186/s12967-020-02275-2

**Published:** 2020-03-04

**Authors:** Yunlong Hu, Li Tang, Zhengyu Zhu, He Meng, Tingting Chen, Sheng Zhao, Zhenchao Jin, Zhulin Wang, Guangyi Jin

**Affiliations:** 1 The Cancer Research Center, School of Medicine, Shenzhen University, Shenzhen, 518055 China; 2 National Engineering LAB of Synthetic Biology of Medicine, School of Medicine, Shenzhen University, Shenzhen, 518055 China; 3 Guangdong Provincial Key Laboratory of Regional Immunity and Diseases, Department of Pathogen Biology, School of Medicine, Shenzhen University, Shenzhen, 518055 China; 4 Shenzhen Kang Tai Biological Products CO., Ltd, Shenzhen, 518060 China; 5 Key Laboratory of Optoelectronic Devices and Systems of Ministry of Education and Guangdong Province, College of Optoelectronic Engineering, Shenzhen University, Shenzhen, 518055 China; 6 Department of Stomatology, Shenzhen University General Hospital & Shenzhen University Clinical Medical Academy, Shenzhen, 518055 China

**Keywords:** Toll-like receptor 7 agonist, Adjuvant, Hepatitis B Virus (HBV), Vaccine

## Abstract

**Background:**

The global burden of hepatitis B virus (HBV) infection in terms of morbidity and mortality is immense. Novel treatments that can induce a protective immune response are urgently needed to effectively control the HBV epidemic and eventually eradicate chronic HBV infection.

**Methods:**

We designed and evaluated an HBV therapeutic vaccine consisting of a novel Toll-like receptor 7 (TLR7) agonist T7-EA, an Alum adjuvant and a recombinant HBsAg protein. We used RNA-seq, ELISA and hTLR7/8 reporting assays to characterize T7-EA in vitro and real-time PCR to evaluate the tissue-retention characteristics in vivo. To evaluate the adjuvant potential, we administrated T7-EA intraperitoneally in a formulation with an Alum adjuvant and HBsAg in normal and HBV mice, then, we evaluated the HBsAg-specific immune responses by ELISA and Elispot assays.

**Results:**

T7-EA acted as an hTLR7-specific agonist and induced a similar gene expression pattern as an unmodified TLR7 ligand when Raw 264.7 cells were exposed to T7-EA; however, T7-EA was more potent than the unmodified TLR7 ligand. In vivo studies showed that T7-EA had tissue-retaining activity with stimulating local cytokine and chemokine expression for up to 7 days. T7-EA could induce Th1-type immune responses, as evidenced by an increased HBsAg-specific IgG2a titer and a T-cell response in normal mice compared to mice received traditional Alum-adjuvant HBV vaccine. Importantly, T7-EA could break immune tolerance and induce persistent HBsAg-specific antibody and T-cell responses in an HBV mouse model.

**Conclusions:**

T7-EA might be a candidate adjuvant in a prophylactic and therapeutic HBV vaccine.

## Background

Chronic hepatitis B virus (HBV) infection is a major global health problem that affects > 350 million people worldwide. The infection can eventually lead to hepatocellular carcinoma, liver failure, or cirrhosis, and causes ~ 1 million deaths every year [1]. Treatments for chronic HBV typically involve the life-long administration of approved drugs (e.g., nucleotides and interferon-α therapies) that are costly and require patient compliance. Long-term interferon therapy can induce many adverse effects [2]; therefore, interruption to treatment results in unavoidable viral rebound. Less than 10% of patients achieve functional cure, defined as anti-HBs seroconversion [3, 4]. There is thus an urgent need for new treatments that can effectively control the HBV epidemic and eventually eradicate chronic HBV infection.

HBV is a DNA virus that is converted into a covalently closed circular DNA (cccDNA) in the host cell nucleus [5]. Licensed antivirals target HBV reverse transcriptase activity but they fail to eliminate cccDNA. HBV cccDNA elimination is currently only achieved by antiviral immune responses [5]; however, HBV-specific T cells are scarce and are functionally impaired in the context of chronic HBV infection most likely due to high amounts of circulating viral HBeAg and HBsAg [6]. A therapeutic vaccine that is combined with antivirals might restore a functional T-cell response and achieve anti-hepatitis B seroconversion while minimizing the risk of adverse effects. Numerous therapeutic vaccination strategies have been developed over the past 20 years and some have even entered clinical trials. Unfortunately, all these clinical trials have showed disappointing results [7, 8]. More sophisticated approaches are warranted to break immune tolerance and boost functional HBV-specific immune responses in individuals with a chronic infection.

Many different approaches have been investigated in preclinical models to overcome immune tolerance in chronic HBV infection. One option is to optimize HBV components in the vaccine, such as DNA or peptide vaccines, vector-based or cell-based vaccines, or multi-epitope therapeutic vaccine candidates that sufficiently cover different HBV genotypes have been developed [9]. Another approach is to develop an adjuvant that can break immune tolerance and induce a functional HBV-specific immune response. Indeed, adjuvant technology is one of the leading approaches to developing novel, safe and effective vaccines.

Toll-like receptors (TLR) are a type of pattern recognition receptor (PRR) widely expressed by immune cells. The ability of TLRs to trigger the innate immune system and boost adaptive immunity is well established [10, 11]. One HBV therapeutic vaccine in clinical trial consisting of HBV antigen-antibody (HBsAg-HBIG) complex with Alum can activate TLR3 on immune cells to induce protective HBV-specific immune response [8]. In addition, a TLR9 agonist CpG has been used as an adjuvant to improve the immune control of persistent HBV infection [12]. A TLR7 agonist has also been reported to induce an innate cytokine response that suppresses HBV replication and to shape the adaptive immune response to achieve durable control of HBV infection [13]. Uniquely designed TLR7 agonists showed adjuvant potential in SIV-infected rhesus monkeys treated with an ad26/MVA therapeutic vaccine and in a prophylactic vaccine against *staphylococcus aureus* [14, 15].

However, TLR7/8 agonists are small molecules and have fast metabolism and narrow drug effect window properties leading to inefficient and inconsistent results thus far [16–18]. Here, we aimed to generate a preferable TLR7 agonist that could be used as adjuvant in an HBV therapeutic vaccine. We chemically synthesized a novel TLR7 agonist T7-EA and studied its drug characterizations in vitro and in vivo. We evaluated the potential of T7-EA to be used as an adjuvant in an HBV therapeutic vaccine in terms of breaking immune tolerance and triggering a high quality HBsAg-specific immune response in normal mice and HBV mouse models.

## Materials and methods

### Mice and viruses

Female Balb/c and C57BL/6 mice aged 4-6 weeks old were purchased from the Animal Center in Guangdong Province (Guangdong, China) and housed in a specific pathogen free (SPF) animal area. All animal experiments were performed in accordance with the Declaration of Helsinki and the protocols were approved by the Shenzhen University School of Medicine Animal Studies and Committee. AAV/HBV virus was provided by the Beijing FivePlus Molecular Medicine Institute (Beijing, China). This recombinant virus carries 1.3 copies of the HBV genome (genotype D, serotype ayw) and is packaged in AAV serotype 8 (AAV8) capsids.

### Chemical synthesis

All chemicals were purchased from commercial vendors and used without further purification, unless noted. Anhydrous *N*,N-dimethylmethanamide (DMF) was distilled over CaH_2_. Non-aqueous reactions were carried out under a nitrogen atmosphere in oven-dried glassware. Thin layer chromatography was performed using precoated silica plates (Merck 60 F254, Alum sheets) and visualized by UV light. ^1^H NMR and ^13^C NMR spectra were obtained on a Bruker Avance III 400 MHz NMR spectrometer. The chemical shifts are reported in parts per million (ppm) relative to internal standard TMS at 0 ppm. Mass spectra were obtained on an Agilent 6224 TOF LC-MS system.

### Conjugation of Toll-like receptor 7 agonist to ethacrynic acid

*9*-*(4*-*(aminomethyl)benzyl)*-*8*-*methoxy*-*2*-*(2*-*methoxyethoxy)*-*9H*-*purin*-*6*-*amine (compound****2****)*: 4-((6-amino-8-methoxy-2-(2-methoxyethoxy)-9H-purin-9-yl) methyl) benzonitrile *(compound****1****)* was prepared following previously reported methods [19]. Raney Ni (500 mg) and ammonium hydroxide (3 mL) were added to a solution of compound **1** (1.5 g, 4.23 mmol) in 1,4-dioxane (40 mL). The reaction mixture was subjected to hydrogenation at 3.5 atm, at 50 °C for 7 h. After reaction completion, the mixture was filtered and the filtrate was concentrated under reduced pressure to afford compound **2** (1.38 g, 91%) as a white solid. ESI–MS (positive ion mode): calculated for C_17_H_23_N_6_O_3_ m/z [M + 1] 359.2; found 359.0.

*6*-*amino*-*9*-*(4*-*(aminomethyl)benzyl)*-*2*-*(2*-*methoxyethoxy)*-*9H*-*purin*-*8*-*ol (compound****3****)*: A suspension of compound **2** (358 mg, 1 mmol) in concentrated hydrochloric acid (10 mL) was placed in a round-bottomed flask. The reaction was stirred at room temperature overnight. The mixture was adjusted to pH 9 with 2 N NaOH and further purified by precipitating and washing in water to obtain a white solid (317 mg, 93%). ESI–MS (positive ion mode): calculated for C_16_H_21_N_6_O_3_ m/z [M + 1] 345.2; found 345.0.

*N*-*(4*-*((6*-*amino*-*8*-*hydroxy*-*2*-*(2*-*methoxyethoxy)*-*9H*-*purin*-*9*-*yl)methyl)benzyl)*-*2*-*(2,3*-*dichloro*-*4*-*(2*-*methylenebutanoyl)phenoxy)acetamide (compound****4***, ***T7*-*EA****)*: To a solution of ethacrynic acid (EA, 341 mg, 1.125 mmol), O-Benzotriazole-N,N,N’,N’-tetramethyl- uronium-hexafluorophosphate (HBTU) (427 mg, 1.125 mmol) dissolved in anhydrous DMF (8 mL) was added triethylamine (TEA) (0.42 mL, 3 mmol) and a catalytic amount of 4-dimethylaminopyridine. A solution of compound **3** (344 mg, 1 mmol) in DMF (2 mL) was added dropwise to the reaction mixture and stirred at room temperature for 4 h. After reaction completion, the reaction content was diluted with water (100 mL), then filtered and washed with ice-cold water. The crude material was purified by flash chromatography (5% MeOH/CH_2_Cl_2_) to afford compound **4** (**T7-EA**) as a white solid (475 mg, 76%). ^1^H-NMR (400 MHz, DMSO-*d*_6_) *δ* 9.97 (s, 1H), 8.54 (t, *J* = 6.0 Hz, 1H), 7.34 (d, *J* = 8.6 Hz, 1H), 7.25 (d, *J* = 8.3 Hz, 2H), 7.21 (d, *J* = 8.3 Hz, 2H), 7.10 (d, *J* = 8.6 Hz, 1H), 6.48 (s, 2H), 6.07 (s, 1H), 5.55 (s, 1H), 4.83 (s, 2H), 4.79 (s, 2H), 4.30 (d, *J* = 6.0 Hz, 2H), 4.28-4.22 (m, 2H), 3.62-3.55 (m, 2H), 3.27 (s, 3H), 2.38 (q, *J* = 7.4 Hz, 2H), 1.09 (t, *J *= 7.4 Hz, 3H). ^13^C-NMR (100 MHz, DMSO-*d*_6_) *δ* 195.59, 167.14, 160.31, 155.97, 152.71, 149.88, 149.64, 148.18, 144.42, 138.65, 136.28, 132.97, 129.86, 129.81, 127.98, 127.93, 121.73, 112.39, 98.80, 70.70, 68.40, 65.75, 58.53, 42.62, 42.19, 23.41, 12.84. ESI–MS (positive ion mode): calculated for C_29_H_31_Cl_2_N_6_O_6_ m/z [M + 1] 629.2; found 629.1.

### RNA sequencing (RNA-seq)

Raw264.7 macrophage cells were seeded in 6-well plates in dulbecco’s modified eagle medium (DMEM) with 10% of fetal bovine serum (FBS), at a density of 1 × 10^6^ cells per well. Compounds were added to the cultures at a final concentration of 10 μM. After 24 h of incubation, total RNA was isolated and purified using an RNA Purification Kit (Omega Bio-Tek, Doraville, USA). RNA-seq was performed on a BGI-SEQ 500 platform and analyzed by BGI (BGI, Shenzhen, China).

### TLR7/8 reporter assay

HEK-Blue hTLR7 and hTLR8 reporter cells were purchased from InvivoGen (InvivoGen, San Diego, USA). These cells stably express human TLR7 and TLR8, and a SEAP reporter that can detect TLR7 and TLR8 agonists through NF-κB signaling activation. The cells were maintained in selective DMEM growth medium with 10 μg/mL blasticidin and 100 μg/mL Zeocin™ (InvivoGen, San Diego, USA). After incubation with different doses of SZU-101 (T7) and T7-EA, the cells were tested using a HEK-Blue detection kit, according to the manufacturer’s instructions. The TLR7/8 agonist R848 was used as a positive control. The induction of TLR7/8 activation was visualized and assessed by reading the OD at 620–655 nm.

### In vitro measurements of cytotoxicity and cytokines induction

Human monocyte cell line THP-1 cells (0.5 × 10^4^) were incubated for 24 h with T7-EA at concentrations ranging from 0.01 to 10 μM, cell viability was detected with a Cell Counting Kit-8 (CCK8) (Beyotime, China).

Human peripheral blood mononuclear cells (PBMCs) were isolated from human buffy coats obtained from 3 healthy volunteers, as described previously [20]. PBMCs (2 × 10^6^/mL) were incubated with various compounds for 18 h at 37 °C and 5% CO_2_, and then the culture supernatants were collected. The levels of cytokines (IL-6, IFN-α) in the supernatants were determined by ELISA (BD Biosciences Pharmingen, La Jolla, CA). The minimum cytokine detection level was 15 pg/mL.

Bone marrow-derived macrophages (BMDMs) and bone marrow-derived dendritic cells (BMDCs) were isolated from C57BL/6 mice and grown as previously described [21, 22]. Then, the cells were seeded in 96-well plates at a density of 5 × 10^4^ cells per well. Compounds were added to the cultures at a final concentration ranging from 0.01 to 10 μM. After 22 h of incubation, the culture supernatants were collected and assayed by ELISA for IL-6 induction (BD Biosciences Pharmingen, La Jolla, USA). The minimum IL-6 detection level was 15 pg/mL.

### In vitro hepatocyte metabolic stability assay

The metabolic stability of the test compounds (1 µM test concentration) was determined in freshly isolated Sprague–Dawley rat hepatocytes or cryopreserved primary human hepatocytes. Incubations were performed using 100,000 cells/mL (final incubation volume = 1 mL) in William’s E Medium at 37 °C in a constant temperature incubator shaker. Then, 100 µL aliquots were taken at 0 min (control), 15 min, 30 min, 1 h, 2 h and 4 h. The reactions were quenched with 300 µL acetonitrile containing 200 ng/mL tolbutamide, vortex-mixed, and centrifuged at 14,000 rpm for 20 min. The supernatants were separated and assessed by LC/MS/MS (SHIMADZU LCMS-8030). All incubations were performed in triplicate.

### RNA isolation and real-time PCR

Balb/c mice (n = 3) were injected in the gastrocnemius muscles with 35 nmol T7, T7-EA, R848 or vehicle (10% DMSO in saline) in a 50μL volume. After 1, 3 and 7 days of injection, the muscles were harvested and immediately frozen in liquid nitrogen and stored at − 80 °C. Total RNA was isolated from cells or tissues using TRIzol reagent (Invitrogen, Carlsbad, USA). The RNA samples were further purified using a Qiagen RNeasy Protect Kit (Qiagen, Valencia, USA). Reverse transcription was carried out with 1 μg total RNA using SuperScript III (Invitrogen, Carlsbad, USA). Serum HBV DNA was extracted from 100 uL of serum and measured following the manufacturer’s instructions (Qiagen, Hilden, Germany). The cDNA and HBV DNA samples were run in duplicate and quantified by real-time PCR (Bio-rad CFX96, Hercules, USA) using the following primer sets: HBV forward 5′CACATCAGGATTCCTAGGACC3′, reverse 5′GGTGAGTGATTGGAGGTTG3′; β-actin forward 5′ GGCTGTATTCCCCTCCATCG3′, reverse 5′ CCAGTTGGTAACAATGCCATGT3′; IL-4 forward 5′ATGGATGTGCCAAACGTCCT3′, reverse 5′AAGCACCTTGGAAGCCCTAC3′, CCL2 forward 5′TCAGCCAGATGCAGTTAACG3′, reverse 5′CTCTCTTGAGCTTGGTGACA3′; CCL4 forward 5′GCAACACCATGAAGCTCTGC3′, reverse 5′CCATTGGTGCTGAGAACCCT3′; TNF-α forward 5′CAAAATTCGAGTGACAAGCCTG3′, reverse 5′GAGATCCATGCCGTTGGC3′. β-actin was used as an internal control for sample loading and normalization. The data were analyzed using the comparative Ct method, where Ct is the cycle number at which the fluorescence first exceeds the threshold. The ΔCt values from each cell line were obtained by subtracting the Ct values for β-actin from the Ct values for the sample. One difference of Ct value represents a 2-fold difference in the level of mRNA. The mRNA level was expressed as a percentage with respect to the control.

### AAV/HBV infection

C57BL/6 mice were injected with the indicated amounts of recombinant virus (diluted to 200 µL with phosphate-buffered saline) via the tail vein. The mice were then bled retro-orbitally at different time points to monitor HBV surface antigen (HBsAg), HBs Antibody (HBsAb) and HBV genomic DNA levels in the serum. HBsAg and HBsAb levels were measured using an ARCHITECT Detection Kit (Abbott, Chicago, USA) following the manufacturer’s instructions.

### In vivo adjuvanticity studies using the HBV vaccine

Purified HBsAg antigen and Alum adjuvant were supplied by Kangtai Biological Products CO., Ltd. (Shenzhen, CHN). For in vivo adjuvanticity studies, uninfected Balb/c and AAV/HBV-infected C57BL/6 mice were immunized intraperitoneally with 2 μg HBsAg mixed with 100 nmol T7, T7-EA or R848 with or without Alum adjuvant at day 0, 14 and 28. Mice immunized with normal saline or HBsAg mixed with vehicle served as controls. Serum was obtained at different time points for HBsAg-specific antibody detection, and splenocytes were isolated to detect the HBsAg-specific T-cell response by Elispot. Anti-HBsAg IgG antibody levels were measured by ELISA with the ARCHITECT Detection Kit. Anti-HBsAg IgG1 and IgG2a were measured as previously described [23]. Briefly, each ELISA plate contained a titration of a previously quantitated serum to generate a standard curve. The titer of this standard was calculated as the highest dilution of serum that gave an absorbance reading that was double the background. The various sera samples were used at a 1:1000 dilution. The results are expressed in units per mL, calculated based on the units/mL of the standard serum. The mean ± SD of five animals in each group are presented. For Elispot assay, 3 × 10^5^ cells/well splenocytes were added in duplicate to 96-well plates coated with anti-mouse IFN-γ (BD Biosciences Pharmingen, La Jolla, USA). Then, 20 μg/mL HBsAg protein was added per well and incubated for 18 h at 37 °C. The number of spot-forming cells was determined using an Axioplan 2 imaging system (Zeiss, Jena, Germany).

### In Vivo cell depletion in adjuvanticity studies

Mouse antibodies (Abs) against CD4 (GK1.5), Ly6C cells and respective isotype control were purchased from Bio X cell (Bio X cell, West Lebanon, USA). Abs were injected 1 day before vaccine administration and repeated every 4 days to maintain cell depletion. Cell depletion was confirmed by flow cytometry on peripheral blood samples 2 days after Ab injection. HBsAg (2 μg) mixed with 100 nmol T7-EA and Alum adjuvant was used for immunization at day 0, and HBsAg mixed with Alum adjuvant alone was used as a control. The mice were sacrificed 7 days after immunization and splenocytes were isolated to detect the HBsAg-specific T-cell response by Elispot, as described above.

### Statistical analysis

All the data are expressed as the mean ± SD. Statistical analysis was performed with GraphPad Prism 5 software. Data were analyzed by one-way ANOVA or unpaired two-tailed t tests, and differences were considered statistically significant at *P *< 0.05. *P < 0.05, **P < 0.01. All experiments were performed at least three times with comparable results.

## Results

### Chemical synthesis of T7-EA

**Fig. 1:**
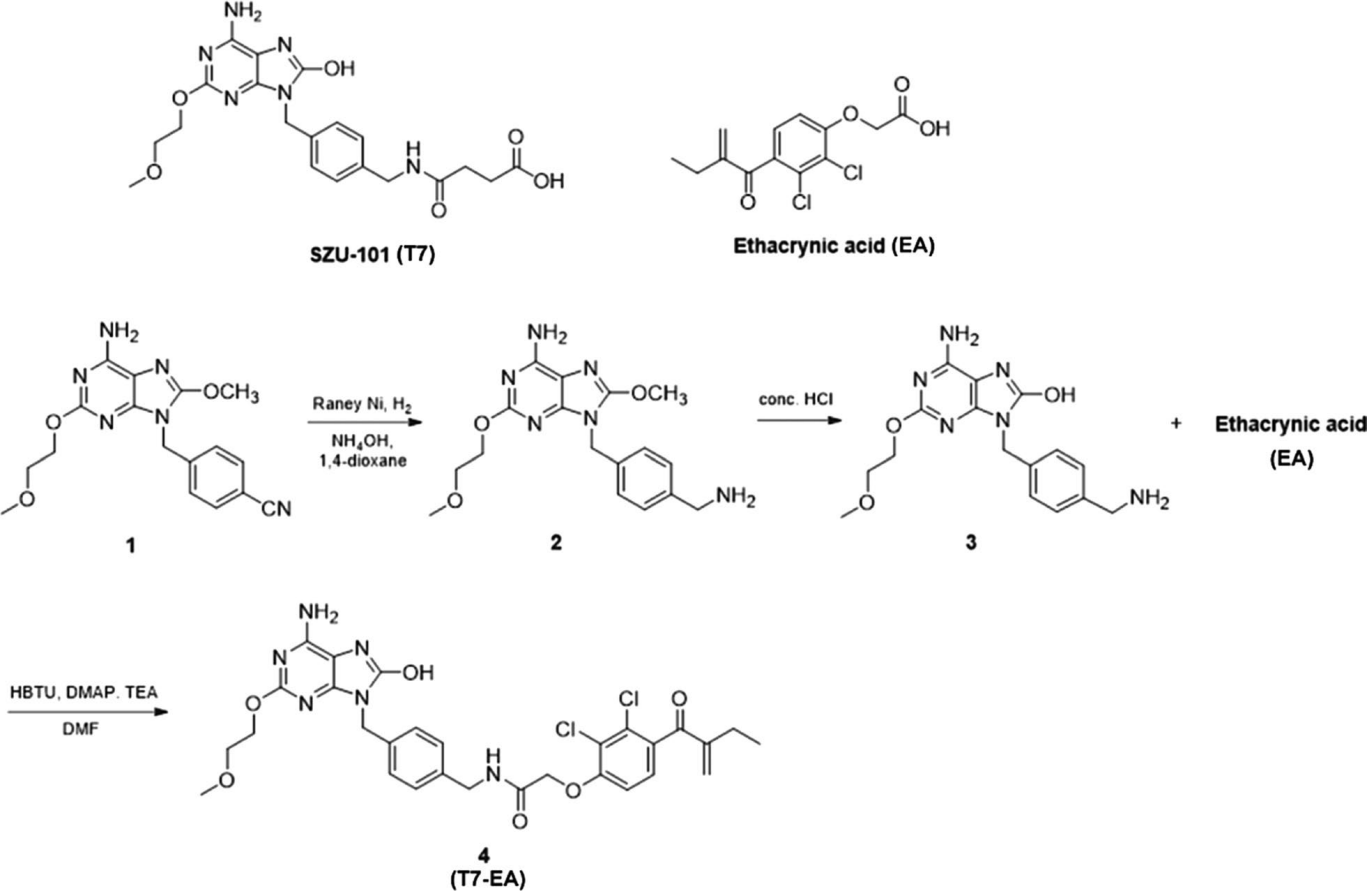
Chemical Synthesis of T7-EA. The molecular structures of SZU-101 (T7) and ethacrynic acid (EA) and the chemical synthesis of the T7-EA conjugate

The synthetic route of T7-EA (compound **4**) is outlined in Fig. 1. We began by (6-amino-8-methoxy-2-(2-methoxyethoxy)-9H-purin-9-yl)methyl benzonitrile (compound **1**), a TLR7 agonist intermediate previously described by our lab [19]. Compound **2** was obtained via reduction of the 9-benzylnitrile group of intermediate **1**. Demethylation of compound **2** with concentrated hydrochloric acid provided 8-hydroxyl analogue **3**. T7-EA (**4**) was synthesized by coupling the TLR7 agonist intermediate **3**, with ethacrynic acid (EA) activating by HBTU. The SZU-101 (T7) pharmacophore and the EA electrophilic α, β-unsaturated ketone moiety were kept.

### Activity evaluation of T7-EA with RNA-seq

**Fig. 2:**
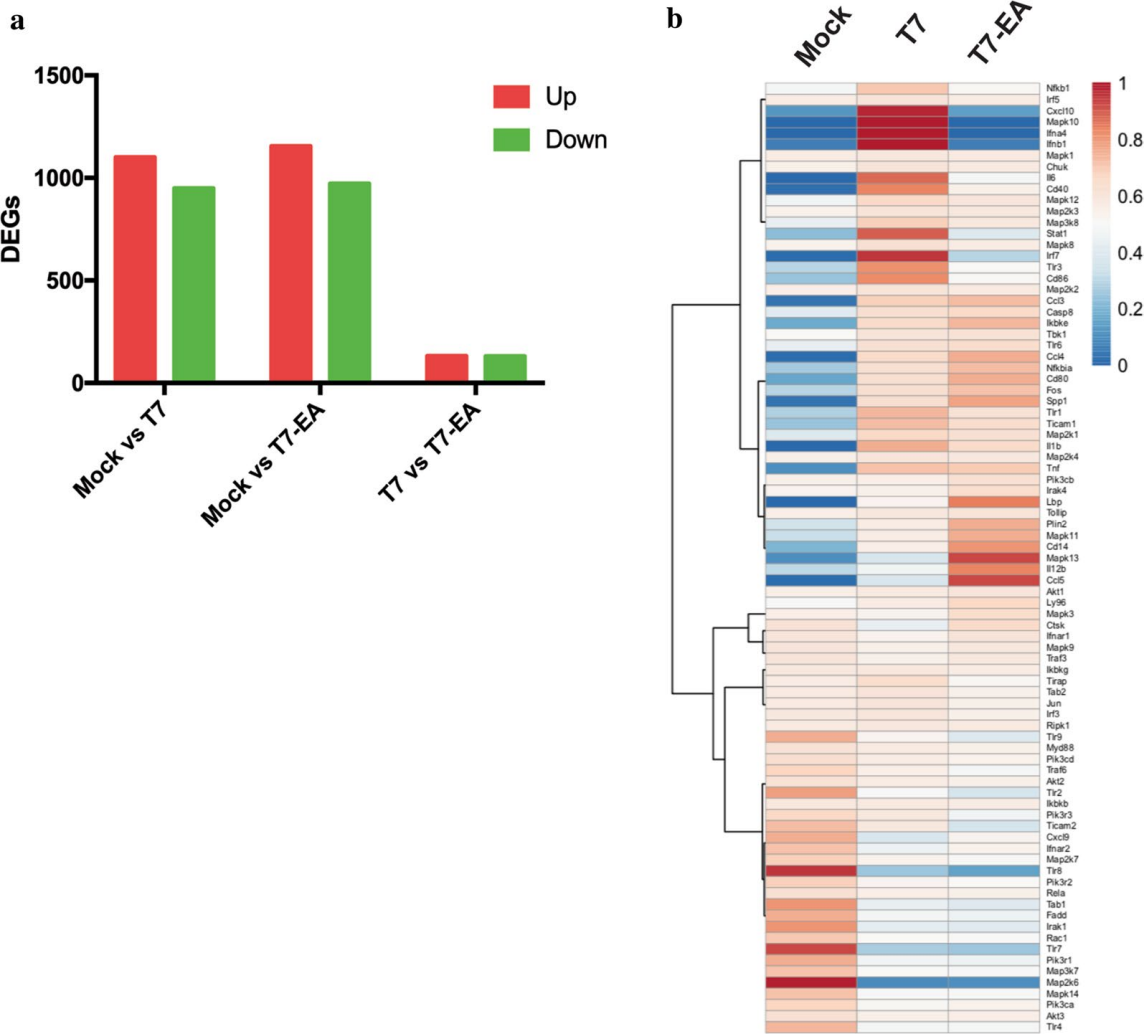
Evaluation of T7-EA activity by RNA-sequencing in Raw264.7 cells. **a** DEGs between T7-EA and T7 treatments. 1 × 10^6^ Raw 264.7 cells were treated with 10 μM T7 or T7-EA for 24 h. Then, total RNA was extracted and purified for RNA-seq, and the DEGs among Mock, T7 and T7-EA groups were counted. **b** Gene expression patterns based on genes involved in the TLR signaling pathway after T7-EA or T7 treatment. All the genes in the TLR signaling were extracted from *PathCards* (https://pathcards.genecards.org), and the gene expression was analyzed in Mock, T7 and T7-EA treatment groups. DEGs, differentially expressed genes; T7, SZU-101

To evaluate T7-EA activity, we treated Raw 264.7 macrophages that overexpress TLR7 and TLR8, with 10 μM T7 and T7-EA for 24 h. We then performed RNA-seq to identify changes in gene expression in response to treatment. We found 130 upregulated genes and 132 downregulated genes (with an absolute fold-change > 2) between Raw 264.7 cells treated with T7-EA and Raw 264.7 cells treated with T7 (Fig. 2a, Additional file 1: Table S1). To compare TLR signaling stimulation upon T7-EA and T7 treatment, we analyzed all the known genes involved in TLR signaling (identified from *PathCards*; https://pathcards.genecards.org) in our RNA-seq dataset. We found similar TLR gene expression patterns between T7-EA and T7 treatments in Raw 264.7 cells: only six genes were differentially expressed with an absolute fold-change > 2. These six genes, Stat1, Ifnb1, Irf7, Cxcl10, Ccl5 and Mapk13 (Fig. 2b, Additional file 2: Table S2), were previously reported as interferon stimulation genes (ISGs) [24]. Further analysis showed that more ISGs not involved in TLR signaling were downregulated by T7-EA compared to T7 (Additional file 3: Fig. S1, Additional file 4: Table S3). These results suggest that while T7-EA has a similar biological function as unmodified T7 in terms of stimulating TLR signaling genes, T7-EA and T7 have differential effects on inducing ISGs in Raw 264.7 cells.

### T7-EA is a TLR7-selective agonist that induces local cytokines and chemokines expression

**Fig. 3:**
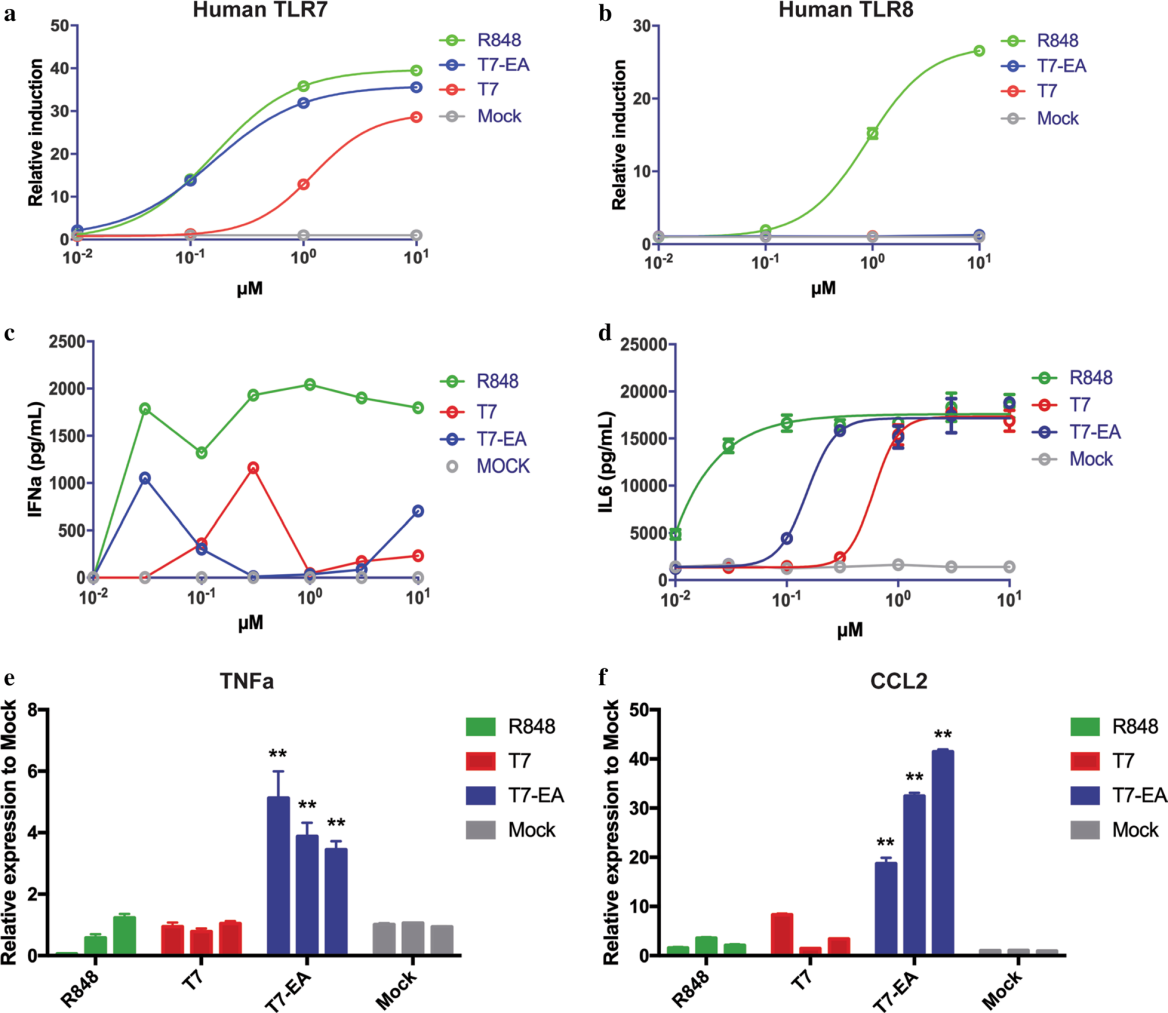
T7-EA is a TLR7-selective agonist that induces local cytokine and chemokine expression. **a**, **b** T7-EA, T7 and R848 were incubated with HEK293 cells expressing either human TLR7 or TLR8 at concentrations ranging from 0.01 to 10 μM. The HEK-Blue 293 reporter system is based on HEK293 cells that are stably transfected with hTLR7 or hTLR8 and a SEAP reporter. SEAP activity can be used to quantify NFκB activation. **c** Human PBMCs (2 × 10^6^/mL) were incubated with T7-EA and T7 at concentrations ranging from 0.01 to 10 μM for 18 h. The levels of IFNα in the culture supernatants were determined by ELISA. **d** BMDMs (0.5 × 10^6^/mL) derived from C57BL/6 mice were incubated with T7-EA, T7 and R848 at concentrations ranging from 0.01 to 10 μM for 22 h. IL-6 levels in the supernatants were measured by ELISA. **e**, **f** Balb/c mice (n = 3) were injected with 35 nmol T7, T7-EA, R848 or vehicle (10% DMSO in saline) in a 50-μL volume in the gastrocnemius muscles. Then, 1, 3 and 7 days after injection, the muscles were harvested. RNA was isolated from muscles, and TNF-α and CCL2 expression levels at the site of injection were determined by real-time-PCR. A student’s *t* test was used for data analysis. The data represent the mean ± SD of triplicates and are representative of three independent experiments. ** *P *< 0.01. T7, SZU-101; BMDMs, bone marrow derived macrophages

TLR7/8 signaling activation will lead to the activation of NF-κB signaling in a MyD88-dependent manner, to clarify the specificity of T7-EA on TLR7 and TLR8, we performed an NF-κB reporter assay using HEK-Blue hTLR7 and hTLR8 reporter cell lines. We found that T7-EA induced more potent SEAP reaction than T7 in HEK-Blue hTLR7 reporter cells, but no activity was observed in inducing SEAP reaction in HEK-Blue hTLR8 reporter cells, suggesting that T7-EA is a specific TLR7 agonist that is more potent than T7 (Fig. 3a, b). We next assessed the immunological effects of T7-EA in PBMCs. We treated PBMCs from 3 healthy donors with T7-EA and T7 at different concentrations for 18 h, and determined the levels of IFN-α and IL-6 in the supernatants. Here, we found that T7-EA showed differential capacity to induce IFN-α and IL-6 expression than T7. Specifically, we observed higher levels of IFN-α expression induced in response to 30 nM T7-EA treatment compared to T7 treatment (Fig. 3c), but higher levels of IL-6 expression induced in response to 3 and 10 μM T7-EA treatment compared to T7 treatment in human PBMCs (Additional file 5: Fig. S2A). These data are consistent with the RNA-seq analysis that showed that T7-EA and T7 treatments induced similar gene expression profiles in genes involved in TLR signaling but showed different activities in inducing ISGs.

We also isolated BMDMs and BMDCs from C57BL/6 mice, and then cultured and stimulated the cells with different concentrations of T7-EA, T7 and R848. The three agonists induced IL-6 expression in a dose-dependent manner in BMDMs and BMDCs: R848 was the most potent agonist with an EC_50_ < 30 nM, while the EC_50_ of T7-EA was ~ 200 nM and the EC_50_ of T7 was ~ 1000 nM (Fig. 3d, Additional file 5: Fig. S2B).

Accumulating evidence suggests that tissue retention is an indispensable characteristic for TLR7 agonist function as a vaccine adjuvant [25, 26]. To test the activity of T7-EA in vivo, we injected 35 nmol T7-EA, T7, R848 or normal saline in the gastrocnemius muscles [intramuscular (i.m.)] of Balb/c mice and then performed real-time PCR with the muscles to detect local cytokine and chemokine expression at day 1, 3 and 7. We found that T7-EA could induce significantly increased TNF-α, CCL2 and CCL4 expression for up to 7 days after the i.m. injection compared to the T7-injected and R848–injected groups (Fig. 3e, f, Additional file 5: Fig S2C–F). These results confirm that T7-EA is a TLR7-selective agonist and can induce local cytokine and chemokine expression.

### T7-EA increases HBsAg-specific Th1 immune responses in a CD4^+^ T cell- dependent manner

**Fig. 4:**
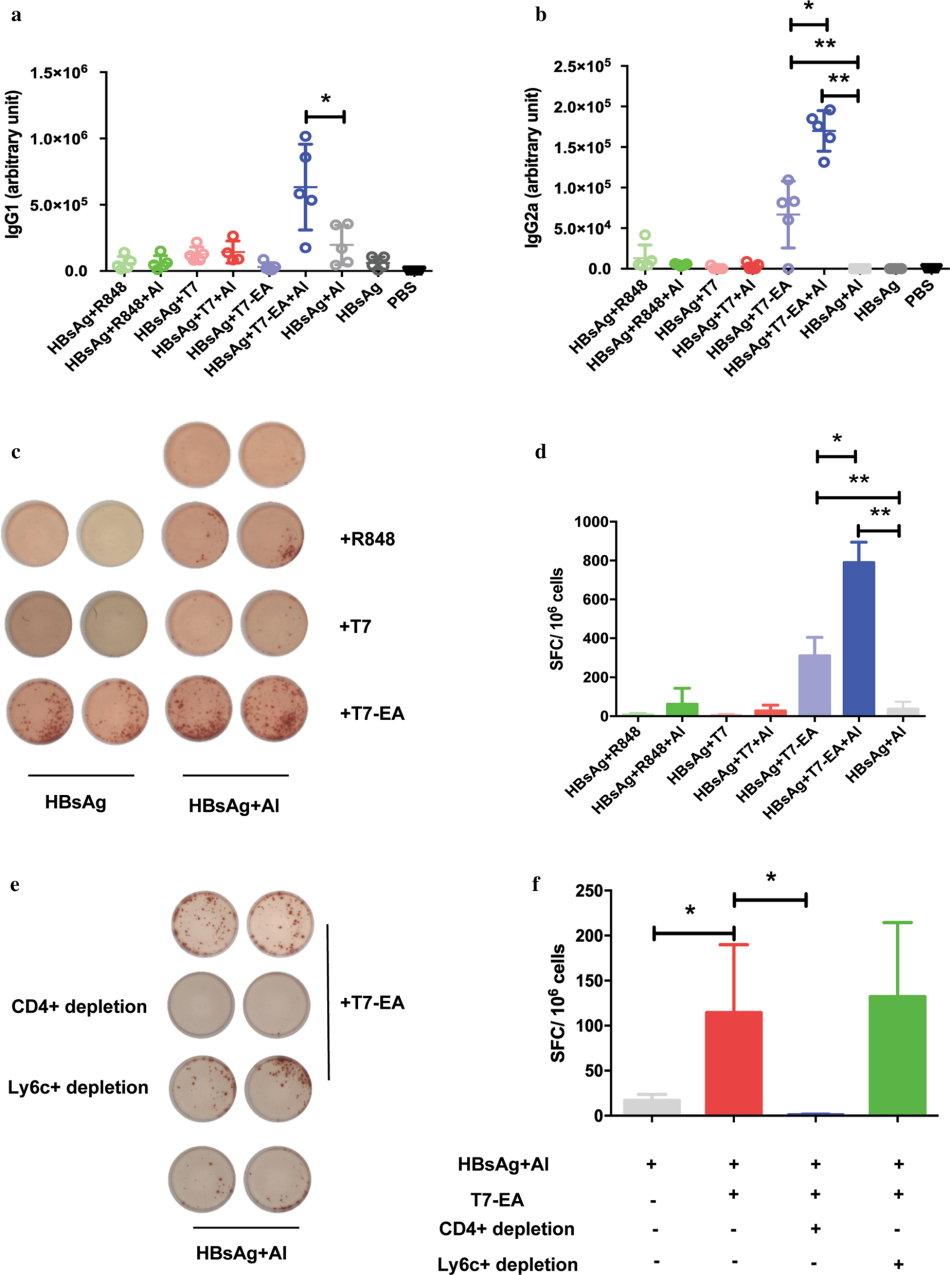
T7-EA increases HBsAg-specific Th1 immune responses in CD4^+^ T cell dependent manner. **a**, **b** Groups of Balb/c mice (n = 5 per group) were immunized intraperitoneally with 2 μg HBsAg mixed with 100 nmol T7, T7-EA or R848 at day 0, 14 and 28. HBsAg specific IgG1 and IgG2a were measured by ELISA in serum obtained 7 days after the third immunization. **c**, **d** Splenocytes were isolated 7 days after the third immunization, and HBsAg-specific T cells were detected by Elispot assay. The cells were cultured at a density of 3 × 10^5^ cells/well with 20 μg/mL HBsAg in a 96 well pre-coated Elispot plate for 18 h. **e**, **f** Groups of Balb/c mice (n = 4 per group) were immunized intraperitoneally with 2 μg HBsAg mixed with 100 nmol T7-EA and Alum adjuvant at day 0. CD4^+^ or Ly6c^+^ cells were depleted by antibody treatment. Splenocytes were isolated 7 days after the immunization. Elispot assay was used for HBsAg-specific T-cell detection. The cells were cultured at a density of 3×10^5^ cells/well with 20 μg/mL HBsAg in the 96 well pre-coated Elispot plate for 18 h. A student’s *t* test was used for data analysis. The data represent the mean ± SD. * *P *< 0.05

TLR7 agonists are efficient adjuvants in inducing an antigen-specific Th1 response, and synergistic effects have been reported when they are administrated with an Alum adjuvant [15, 26, 27]. We thus used T7-EA as an adjuvant in an HBV vaccine. We administered T7-EA intraperitoneally in a formulation with an Alum adjuvant and a recombinant HBsAg protein in normal Balb/c mice on day 0, 14 and 28, T7 and a TLR7/8 agonist R848 were used as controls. We then evaluated the HBsAg-specific humoral and cellular immune responses on day 35. We found that T7-EA could induce a high titer of HBsAg-specific IgG2a in serum, this effect was absent with the traditional Alum-adjuvant HBV vaccine, and we observed a further increase in HBsAg-specific IgG1 and IgG2a titers in the T7-EA plus Alum group compared to all other groups (Fig. 4a, b). In terms of the cellular immune response, T7-EA could induce a high quality HBsAg-specific T-cell response, as evidenced by the production of more spot forming cells (SFC) compared to other groups, and a further increase in the HBsAg-specific T-cell response was observed in the T7-EA plus Alum combination group compared to all other groups (Fig. 4c, d). R848 and T7 had no effect on inducing HBsAg-specific T-cell response when used as adjuvants in HBV vaccine. In addition, we didn’t observe significant cytotoxicity of T7-EA on THP-1 cells and side-effects of T7-EA used as an adjuvant in vivo evidenced by spleen/body ratio (Additional file 6: Fig. S4).

From these experiments, the best performance formulation seemed to consist of 100 nmol T7-EA, 2 μg HBsAg and Alum adjuvant as a potential HBV therapeutic vaccine for further analysis. Because other reports show that CpG-ODN and 3 M-052 can induce cytotoxic lymphocyte activity via a T-helper cell-independent mechanism [23, 28], we tested whether the effect of T7-EA on the HBsAg-specific T-cell response was regulated by a T-helper cell-independent mechanism. We performed cell depletion experiments with CD4^+^ or Ly6c^+^ antibody treatment and found that CD4^+^ T-cell depletion abrogated the HBsAg-specific T-cell response in our HBV therapeutic vaccine, but Ly6c^+^ cell depletion showed no significant effect (Fig. 4e, f). These results imply that T7-EA used as an adjuvant can induce a high quality HBsAg-specific immune response in a CD4^+^ T cell-dependent manner.

### T7-EA induces a high-quality immune response in an HBV mouse model

**Fig. 5:**
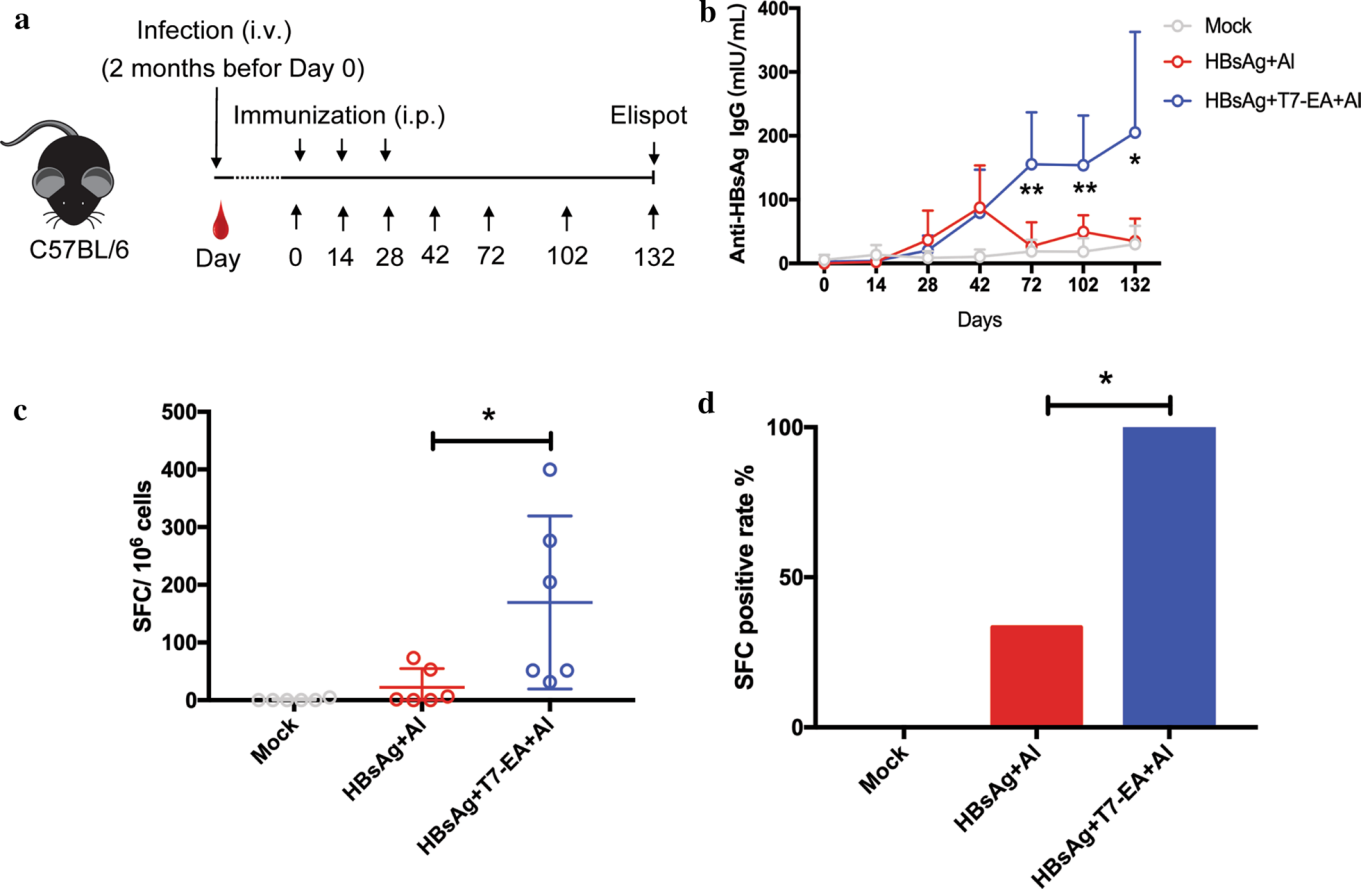
T7-EA induces a high quality immune response in an HBV mouse model. **a** The experimental procedure for the HBV therapeutic vaccine study. **b** Groups of HBV mice (n = 6 per group) were immunized intraperitoneally with HBV therapeutic vaccine consisting of T7-EA, Alum adjuvant and a recombinant HBsAg protein at day 0, 14 and 28. Normal saline and a traditional HBV vaccine were used as controls. HBsAg-specific IgG levels in the serum were measured by ELISA at the indicated time points. **c** HBV mice were sacrificed at day 132 and splenocytes were isolated; then, HBsAg-specific T cells were detected by Elispot assay. The cells were cultured at a density of 3 × 10^5^ cells/well with 20 μg/mL HBsAg in a 96-well pre-coated Elispot plate for 18 h. SFC in 1 × 10^6^ cells are shown. **d** HBsAg-specific T-cell positive rates. SFC were detected with Elispot and the cells cultured without HBsAg stimulation were used as a negative control. SFC in experimental wells with two times more than the negative control wells were regarded as being HBsAg-specific T-cell positive. SFC, spot-forming cells

During chronic HBV infection, the HBV induces immune tolerance in part by secreting extremely high levels of HBsAg and HBeAg: this effect likely skews T-cell function. A therapeutic vaccine should, therefore, overcome immune tolerance and induce a protective immune response for HBV clearance [29]. We further tested the efficiency of our newly designed HBV therapeutic vaccine, consisting T7-EA adjuvant, Alum adjuvant and a recombinant HBsAg protein, in breaking immune tolerance in an HBV mouse model. The HBV mouse model was generated using a recombinant AAV carrying the HBV genome (AAV/HBV) that was previously reported as an appropriate model for developing novel therapies for chronic HBV infection [30]. Two months after tail vein injection of the AAV/HBV virus, we administered our HBV therapeutic vaccine intraperitoneally on days 0, 14 and 28. We then detected HBsAg, HBsAb and HBV genomic DNA in the serum at different time points before sacrificing the mice to evaluate the HBsAg-specific T-cell response on day 132 (Fig. 5a). The HBV therapeutic vaccine could induce 3-sixfold increase and persistent HBsAb response compared to a traditional HBV vaccine from days 72 to days 132 (Fig. 5b), and a significant increased HBsAg-specific T-cell response. Notably, all the mice that received the HBV therapeutic vaccine achieved a HBsAg-specific T-cell-positive response (Fig. 5c, d). We also noted significant downregulation of HBV DNA copies and HBsAg titers in mice receiving the HBV therapeutic vaccine compared to those receiving normal saline, but no significant difference between mice receiving the HBV therapeutic vaccine and traditional vaccine (Additional file 7: Fig. S3A, B). These data support that our new HBV therapeutic vaccine can break immune tolerance and induce high quality HBsAg-specific immune responses in an HBV mouse model.

### T7-EA shows fast metabolism and low toxicity on hepatocyte

To test the metabolic stability of T7-EA, an in vitro hepatocyte metabolic stability assay was used. The elimination half-lives in rat and human primary hepatocytes were 3.1 h and 1.5 h, and the intrinsic clearance rates of the liver were 1217.0 and 1197.1 mL/h/Kg, respective (Additional file 8: Table S4). These data demonstrated that T7-EA has a fast metabolism and low toxicity.

## Discussion

TLRs 7/8 are promising targets in the design and development of small-molecule immunomodulators serving as vaccine adjuvants and antiviral/anticancer agents [31–33]. In this study, we aimed to generate a preferable TLR7 agonist that could be used as adjuvant in an HBV therapeutic vaccine. we chemically synthesized a novel TLR7 agonist, T7-EA by conjugating our reported TLR7 agonist SZU-101 (T7) to ethacrynic acid (EA) [34], the function group of T7 and the α, β-unsaturated ketone group of EA were kept. We then performed RNA-seq to evaluate T7-EA activity in Raw 264.7 macrophages that overexpress TLRs. Here, T7-EA retained the function exhibited by unmodified T7 in terms of stimulating similar genes involved in TLR signaling. We did note, however, significant differences between T7-EA and T7 treatments in terms of ISG induction. We then moved our analyses to hTLR7 and hTLR8 reporter cell lines, primary immune cells and in vivo model to characterize T7-EA. We found that T7-EA is an hTLR7-specific agonist and is more potent in inducing IFN-α and IL-6 expression compared to T7. In addition, T7-EA exhibited tissue-retaining activity and could stimulate local cytokine and chemokine expression for up to 7 days. To evaluate the adjuvant capacity, we administered T7-EA intraperitoneally in a formulation with Alum adjuvant and a recombinant HBsAg protein. T7-EA could induce Th1-type immune responses, as evidenced by an increased HBsAg-specific IgG2a titer and T-cell response compared to traditional HBV vaccine in normal mice. Importantly, T7-EA could break immune tolerance and induce persistent, high quality HBsAg-specific antibody and T-cell responses in HBV-infected mice. These data thus suggest that T7-EA might be used as an adjuvant in a prophylactic and therapeutic HBV vaccine.

The small-molecule characteristics of TLRs 7/8 agonists renders them easy to synthesize and modify, but their fast metabolism and narrow drug effect window properties has led to inefficient and inconsistent results thus far [16–18]. Therefore, several strategies have been devised to optimize TLR 7/8 agonists, such as conjugation with HIV-1 Gag protein for vaccine development, synergy with CD40 and CpG ODN in augmenting immune responses and the invention of novel imidazoquinolines for adjuvant application [16, 35–39]. In this study, we chemically conjugated a TLR7 agonist to EA: EA contains a α, β-unsaturated ketone group that can automatically bind to thiol-containing proteins, such as LEF1 [40]. There is an abundant resource of thiol-containing proteins in cells, tissues and body fluids, meaning that T7-EA with a α, β-unsaturated ketone group should possess new drug activity [41]. In our system, T7-EA acquired the exo-methylene group which we found enabled T7-EA to automatically bind to sulphur proteins, such as GSH, GSH-T7-EA [M + 1] 936.2. We propose that the α, β-unsaturated ketone group might underlie why T7-EA has different pharmacodynamic and pharmacokinetic characteristics compared to unmodified T7. TLR7 signaling activation in early and newly formed endosomes is linked to type I interferon synthesis [42], whereas TLR7 signaling activation in late-stage endosomes induces cytokines (such as TNF-α and IL-6) in a NF-κB-dependent manner [42]. T7-EA showed more potent TLR7 activation activity compared to T7, but we observed significantly different doe-dependent activity in inducing NF-κB-associated genes and ISGs. To clarify the mechanism of the different activity between T7-EA and T7 in inducing NF-κB-associated genes and ISGs, we think that the distribution of T7-EA and T7 in endosomal vesicles across different stages is worth exploration.

TLR7/8 activation leads to Th1 cytokine production, MHC and co-stimulatory molecule upregulation and dendritic cell (DC) maturation. Thus, TLR7/8 agonists are able to drive T-cell-mediated and B-cell-mediated adaptive immune responses [43, 44]. Local DC cell activation is critical for initiating adaptive immune responses; however, reported pharmacokinetic studies in humans, in which imiquimod solution was injected subcutaneously, showed that the serum levels were detectable within 5 min post-dosing (Tmax: ~ 30 min) and > 95% of the imiquimod was eliminated after 12 h [18]. The short half-life of imiquimod at the injection site is likely suboptimal for local DC activation. Thus, a novel adjuvant (Alum/TLR7) formed by attaching a TLR7 agonist to Alum has been designed [15]. Alum/TLR7 shows better local stimulation effects and adjuvant capacity compared to Alum and T7 alone. Alum/TLR7 is currently in phase I clinical development and has been tested in several disease models, such as in the context of staphylococcus, anthrax, meningococcal meningitis infections [15, 27, 45–47]. Here, we generated a coupled molecule T7-EA with local retaining activity and evaluated its potential as an adjuvant in an HBV therapeutic vaccine. T7-EA stimulated a robust HBsAg-dependent Th1 response, as determined by IgG2a induction and T-cell responses in normal mice. We also found a synergistic effect in the T7-EA and Alum combination group, which induced a further increase in the HBsAg-specific T-cell response compared to all other experimental groups. Further studies showed that T7-EA could break immune tolerance and induce persistently high quality HBsAg-specific antibody and T-cell responses in an HBV mouse model. Compared to Alum/TLR7, T7-EA is a small molecule easier to synthesize, store and use, and has a more stable chemical structure. However, a comparison of the adjuvant capacity between Alum/TLR7 and T7-EA is now needed.

## Conclusions

In summary, we have generated a novel TLR7 agonist known as T7-EA, which has promising pharmacodynamic and pharmacokinetic characteristics. We propose that T7-EA might be used as an adjuvant in HBV therapeutic vaccines as it can break immune tolerance and induce high quality HBsAg-specific immune responses in normal and HBV mouse models. Further studies are now needed to confirm the potential of T7-EA as an adjuvant in HBV therapeutic vaccines in other models and in clinical practice. We believe that T7-EA is a candidate for application as an adjuvant in prophylactic and therapeutic hepatitis B vaccines.

## Supplementary information

**Additional file 1: Table S1.** Complete list of DEGs in Raw 264.7 cells treated with T7-EA and T7. All genes had an absolut fold-change (FC) >2 and a false discovery rate (FDR) <0.05 in T7-EA vs T7 group, and passed a low-expression filter.

**Additional file 2: Table S2.** Complete list of genes in the Toll-like receptor signaling pathway. Genes in Toll-like receptor signaling pathway were taken from ***PathCards*** (https://pathcards.genecards.org). Passed a low-expression filter.

**Additional file 3: Figure S1.** T7-EA treatment suppresses ISGs expression compared to T7 treatment in Raw 264.7 cells. Raw 264.7 cells (1 × 10^6^ ) were treated with 10 μM T7 or T7-EA for 24 h; then, total RNA was extracted and purified for RNA-seq. A list of ISGs were taken from Schoggins et al. (2011) *Nature* 472: 481-485. All genes had an absolute fold-change >2 and a false discovery rate <0.5 in the T7-EA vs T7 group, and passed a low-expression filter. ISGs, interferon-stimulated genes.

**Additional file 4: Table S3.** Complete list of ISGs significantly downregulated by T7-EA compared to T7. ISGs were taken from Schoggins et al. (2011) *Nature* 472: 481-485. All genes had an absolute fold-change (FC) >2 and a false discovery rate (FDR) <0.05 in T7-EA vs T7 group, and passed a low-expression filter.

**Additional file 5: Figure. S2.** Evaluation of T7-EA activity in primary immune cells and after tissue injection. (A) Human PBMCs (2 × 10^6^/mL) were incubated for 18 h with T7-EA and T7 at concentrations ranging from 0.01 to 10 μM. IL-6 levels in the culture supernatants were determined by ELISA. (B) BMDCs (0.5 × 10^6^/mL) derived from C57BL/6 mice were incubated for 22 h with T7-EA, T7 and R848 at concentrations ranging from 0.01 to 10 μM. The levels of IL-6 in the supernatants were measured by ELISA. (C-F) Balb/c mice (n=3) were injected in the gastrocnemius muscles with 35 nmol T7, T7-EA, R848 or vehicle (10% DMSO in saline) in a 50 μL volume. Then, 1, 3 and 7 days after injection, the muscles were harvested and RNA was isolated. IL4 and CCL4 expression at the injection site were determined by real-time PCR. A student’s *t* test was used for data analysis. The data represent the means ± SD of triplicates and are representative of three independent experiments. ** *P*<0.01. T7, SZU-101; BMDCs, bone marrow derived dendritic cells

**Additional file 6: Figure S4**. Evaluation of adverse effect of T7-EA. (A) THP-1 cells (0.5 × 10^4^) were incubated for 24 h with T7-EA at concentrations ranging from 0.01 to 10 μM. CCK8 was used to assess cell viability. (B) Groups of HBV mice (n=6 per group) were immunized intraperitoneally with HBV therapeutic vaccine consisting of T7-EA, Alum adjuvant and a recombinant HBsAg protein at day 0, 14 and 28. Normal saline and a traditional HBV vaccine were used as controls. On day 35, the mice were sacrificed and the spleen/body weight ratio was calculated.

**Additional file 7: Figure S3.** The effects of vaccines on HBV DNA and HBsAg levels in an HBV mouse. Groups of HBV mice (n=6 per group) were immunized intraperitoneally with an HBV therapeutic vaccine consisting of T7-EA, Alum adjuvant and a recombinant HBsAg protein at day 0, 14 and 28. Normal saline and a traditional HBV vaccine were used as controls. (A) HBV DNA in the serum was detected by real-time PCR at different time points. The data are expressed as relative to HBV DNA copies at day 0 for each mouse. (B) HBsAg levels in the serum was detected by Elisa at different time points. The data are expressed as relative to HBsAg levels at day 0 for each mouse.

**Additional file 8: Table S4.***In vitro* hepatocyte metabolic stability of T7-EA.

## Data Availability

All data generated or analyzed during this study are included in this article.

## Abbreviations

HBV: Hepatitis B virus
TLR: Toll-like receptor
SZU-101 (T7): A novel TLR7 agonist
EA: Ethacrynic acid
T7-EA: A modified TLR7 agonist based on SUZ-101
HBV: Hepatitis B virus
HBsAg: HBV surface antigen
cccDNA: Covalently closed circular DNA
PRR: Pattern recognition receptor
LC–MS: Liquid Chromatograph Mass Spectrometer
TLC: Thin layer chromatography
EMI-MS: Electrospray ionization mass spectrometry
DMF: Dimethylmethanamide
TEA: Triethylamine
SEAP: Secret alkaline phosphatase
OD: Optical density
PBMC: Peripheral mononuclear cells
IL-6: Interleukin-6
IL-4: Interleukin-4
IFN-α: Interferon-α
BMDM: Bone marrow-derived mononuclear
BMDC: Bone marrow-derived dendritic cell
TNF-α: Tumor necrosis factor alpha
CCL2: C-C motif chemokine ligand 2
CCL4: C-C motif chemokine ligand 4
AAV: Adeno-associated virus
Ly6c: Lymphocyte antigen 6 complex
Ab: Antibody
SFC: Spot forming cells
GSH: Glutathione
MHC: Major histocompatibility complex
DEGs: Different expression genes
ISGs: Interferon-stimulated genes
