# Supplementary figures and images for: A novel TLR7 agonist as adjuvant to stimulate high quality HBsAg-specific immune responses in an HBV mouse model
Source: J Transl Med. 2020 Mar 4;18:112. doi: 10.1186/s12967-020-02275-2 (PMC7055022; doi:10.1186/s12967-020-02275-2)

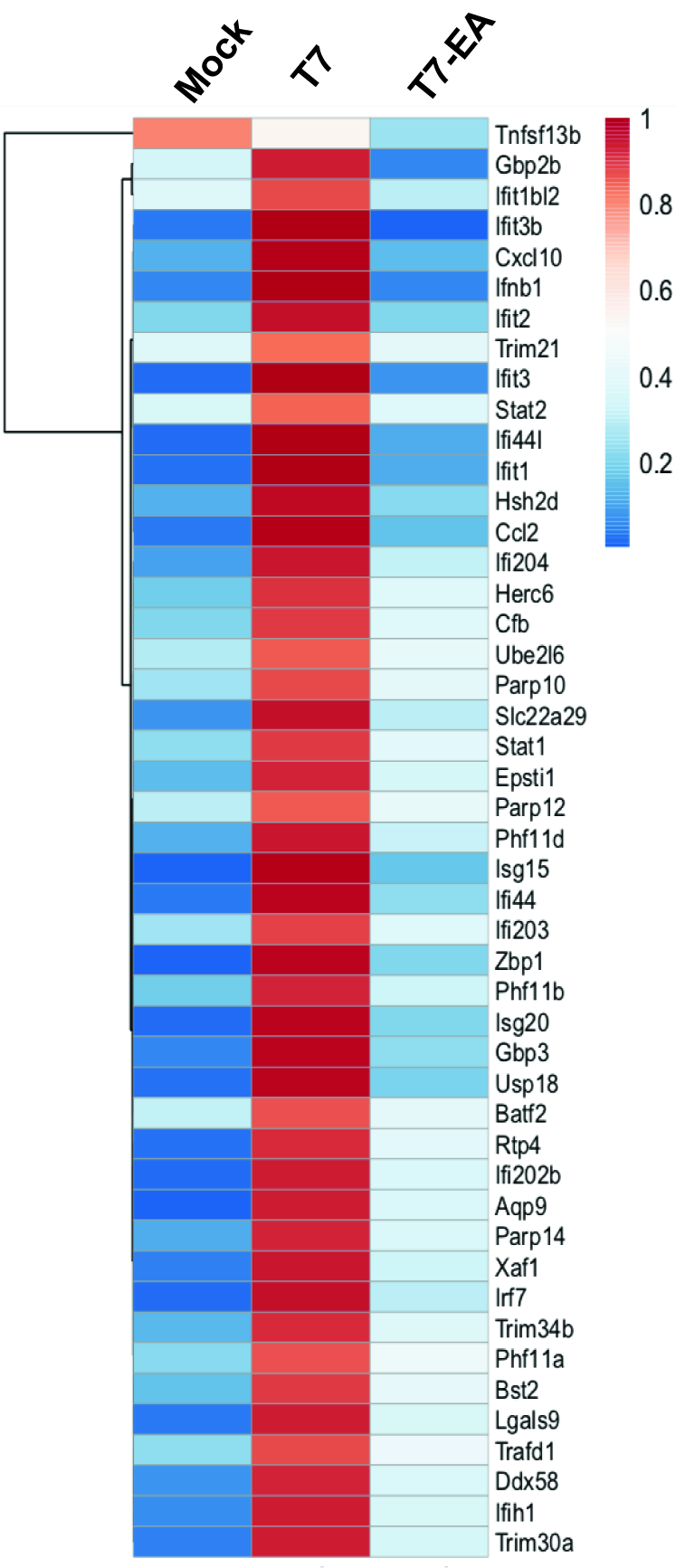

Supplement: Supplementary file 3 — Additional file 3: Figure S1. T7-EA treatment suppresses ISGs expression compared to T7 treatment in Raw 264.7 cells. Raw 264.7 cells (1 × 10^6^ ) were treated with 10 μM T7 or T7-EA for 24 h; then, total RNA was extracted and purified for RNA-seq. A list of ISGs were taken from Schoggins et al. (2011) Nature 472: 481-485. All genes had an absolute fold-change >2 and a false discovery rate <0.5 in the T7-EA vs T7 group, and passed a low-expression filter. ISGs, interferon-stimulated genes. [file 12967_2020_2275_MOESM3_ESM.tif]

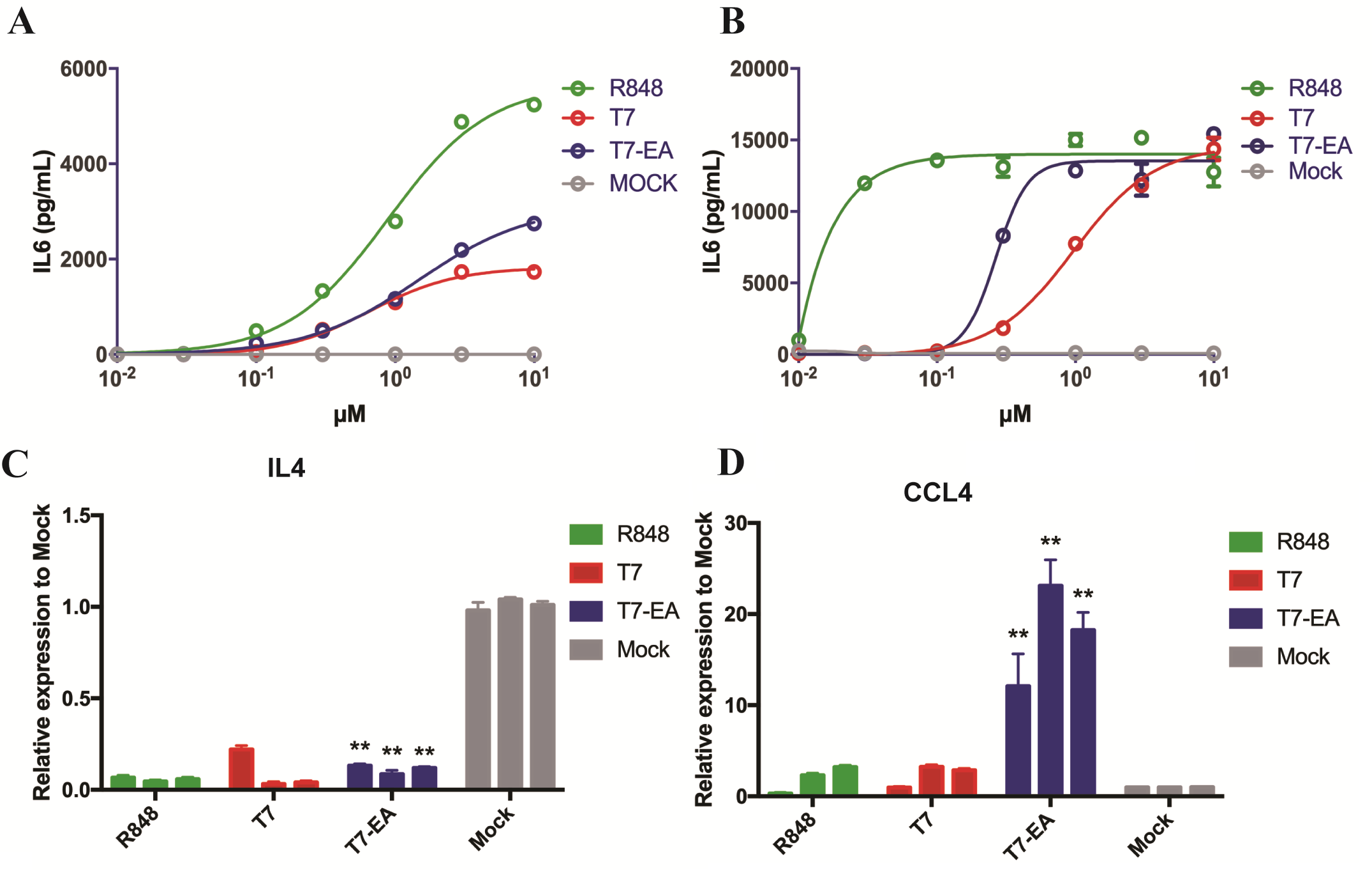

Supplement: Supplementary file 5 — Additional file 5: Figure. S2. Evaluation of T7-EA activity in primary immune cells and after tissue injection. (A) Human PBMCs (2 × 10^6^/mL) were incubated for 18 h with T7-EA and T7 at concentrations ranging from 0.01 to 10 μM. IL-6 levels in the culture supernatants were determined by ELISA. (B) BMDCs (0.5 × 10^6^/mL) derived from C57BL/6 mice were incubated for 22 h with T7-EA, T7 and R848 at concentrations ranging from 0.01 to 10 μM. The levels of IL-6 in the supernatants were measured by ELISA. (C-F) Balb/c mice (n=3) were injected in the gastrocnemius muscles with 35 nmol T7, T7-EA, R848 or vehicle (10% DMSO in saline) in a 50 μL volume. Then, 1, 3 and 7 days after injection, the muscles were harvested and RNA was isolated. IL4 and CCL4 expression at the injection site were determined by real-time PCR. A student’s t test was used for data analysis. The data represent the means ± SD of triplicates and are representative of three independent experiments. ** P<0.01. T7, SZU-101; BMDCs, bone marrow derived dendritic cells [file 12967_2020_2275_MOESM5_ESM.tif]

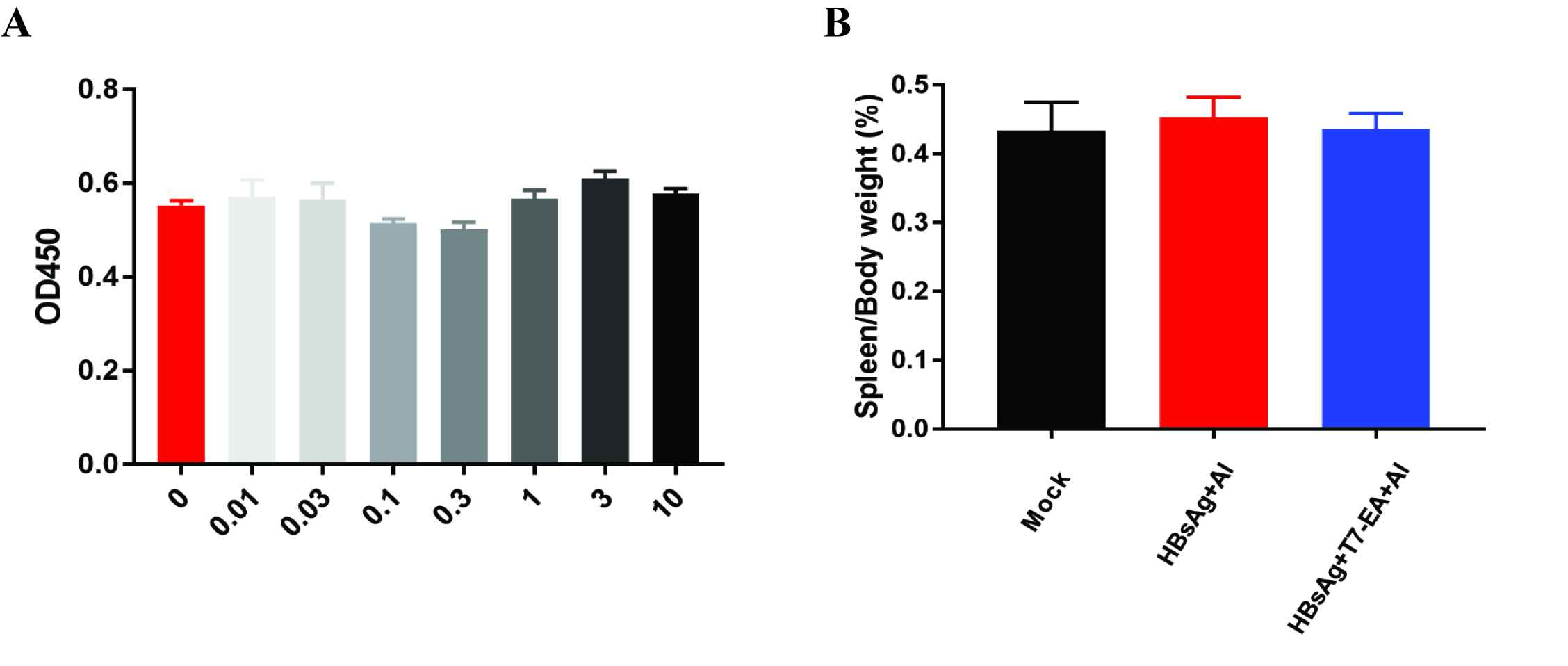

Supplement: Supplementary file 6 — Additional file 6: Figure S4. Evaluation of adverse effect of T7-EA. (A) THP-1 cells (0.5 × 10^4^) were incubated for 24 h with T7-EA at concentrations ranging from 0.01 to 10 μM. CCK8 was used to assess cell viability. (B) Groups of HBV mice (n=6 per group) were immunized intraperitoneally with HBV therapeutic vaccine consisting of T7-EA, Alum adjuvant and a recombinant HBsAg protein at day 0, 14 and 28. Normal saline and a traditional HBV vaccine were used as controls. On day 35, the mice were sacrificed and the spleen/body weight ratio was calculated. [file 12967_2020_2275_MOESM6_ESM.tif]

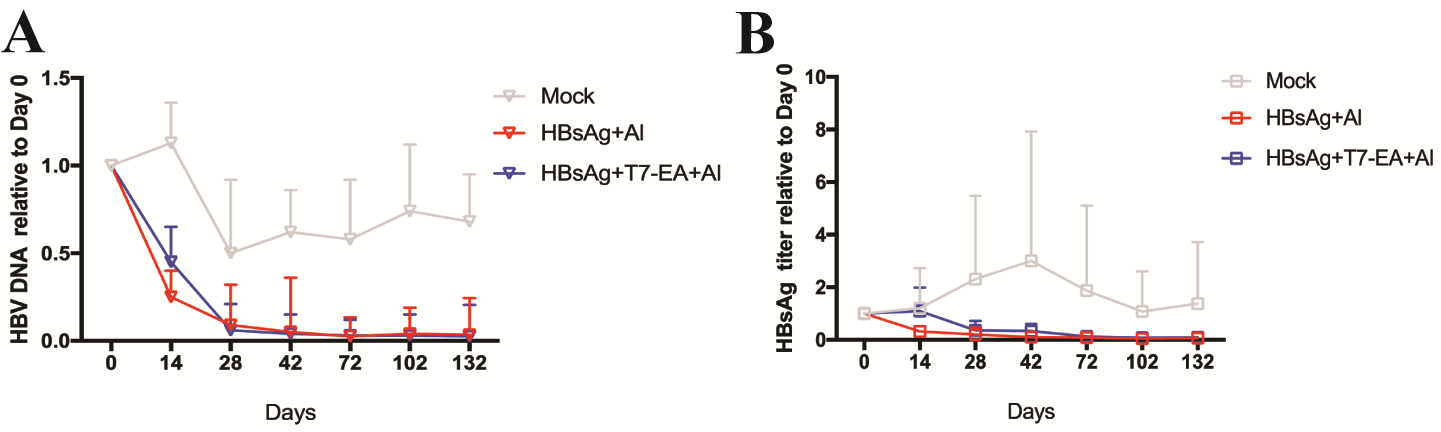

Supplement: Supplementary file 7 — Additional file 7: Figure S3. The effects of vaccines on HBV DNA and HBsAg levels in an HBV mouse. Groups of HBV mice (n=6 per group) were immunized intraperitoneally with an HBV therapeutic vaccine consisting of T7-EA, Alum adjuvant and a recombinant HBsAg protein at day 0, 14 and 28. Normal saline and a traditional HBV vaccine were used as controls. (A) HBV DNA in the serum was detected by real-time PCR at different time points. The data are expressed as relative to HBV DNA copies at day 0 for each mouse. (B) HBsAg levels in the serum was detected by Elisa at different time points. The data are expressed as relative to HBsAg levels at day 0 for each mouse. [file 12967_2020_2275_MOESM7_ESM.tif]
